# Multi-site quantitative benchmarking of Neu5Gc-bearing N-glycans using matrix-matched LC–MS calibration

**DOI:** 10.1093/glycob/cwag088

**Published:** 2026-10-10

**Authors:** Nicholas J DeBono, Edward S X Moh, Jessica Poole, Nicolle H Packer, Christopher J Day, Michael P Jennings, Daniel Kolarich, Christopher Ashwood

**Affiliations:** Institute for Biomedicine and Glycomics, Griffith University, 1 Parklands Dr, Southport, QLD 4215, Australia; ARC Centre of Excellence in Synthetic Biology, Macquarie University, 6 Wally's Walk, Sydney, NSW 2113, Australia; Institute for Biomedicine and Glycomics, Griffith University, 1 Parklands Dr, Southport, QLD 4215, Australia; Institute for Biomedicine and Glycomics, Griffith University, 1 Parklands Dr, Southport, QLD 4215, Australia; ARC Centre of Excellence in Synthetic Biology, Macquarie University, 6 Wally's Walk, Sydney, NSW 2113, Australia; Institute for Biomedicine and Glycomics, Griffith University, 1 Parklands Dr, Southport, QLD 4215, Australia; Institute for Biomedicine and Glycomics, Griffith University, 1 Parklands Dr, Southport, QLD 4215, Australia; Institute for Biomedicine and Glycomics, Griffith University, 1 Parklands Dr, Southport, QLD 4215, Australia; School of Environment and Science, Griffith University, 1 Parklands Dr, Southport, QLD 4215, Australia; Protea Glycosciences Pty Ltd, 93 The Avenue, Wollongong, NSW 2500, Australia

**Keywords:** Glycomics, LCMS, N-glycans, Neu5Gc, sialic acid

## Abstract

*N*-glycolylneuraminic acid (Neu5Gc) has been repeatedly associated with human cancer, but reliable detection in mass-spectrometric studies has remained elusive, but its low abundance has led to inconsistent detection in studies of human samples. To address this, matrix-matched calibration curves, which have been pioneered in proteomics and metabolomics for assessing changes in complex mixtures, were measured of released *N*-glycans at four orders of magnitude dynamic range in defined mixtures, systematically benchmarking Neu5Gc-containing *N*-glycan detection across multiple LC–MS platforms and sites. Orthogonally, the gold-standard analytical method, consisting of fluorescence detection of labelled monosaccharides separated by LC, was applied to the same samples, yielding absolute concentrations of Neu5Gc.

LC–MS demonstrated an extended detection range of three or more orders of magnitude while retaining intact *N*-glycan measurement, improving assay specificity and enabling detection of the variety of Neu5Gc-bearing *N*-glycans. By combining orthogonal dimensions of evidence, including chromatographic separation, isotopic distribution matching, and composition-confirming MS/MS, LC–MS confidently resolved Neu5Gc signals from noise, even at low abundance. In comparison, under the DMB-LC-FLR conditions and sample loading evaluated here, dynamic range was limited to two orders of magnitude, insufficient for detection of Neu5Gc in commercially available pooled human sera. These findings strongly support that the DMB-LC-FLR assay’s specificity and sensitivity were insufficient for Neu5Gc detection in human samples due to noise overwhelming the Neu5Gc signal. By establishing a reusable benchmarking framework for future glycomic studies, we aim to use LC–MS to improve the measurement of Neu5Gc in clinical samples.

## Introduction

Aberrant glycosylation of cancer tissue is a well-established feature of cancer across multiple tumour types (Meany and Chan 2011; Tuccillo et al. 2014; Munkley and Elliott 2016). One repeatedly reported alteration is the presence of *N*-glycolylneuraminic acid (Neu5Gc) (Wang et al. 2023). Neu5Gc has historically been considered to be absent or unexpected in healthy human glycomics and has consequently been omitted from, or treated as an unexpected feature in some human glycomic and glycoproteomic analyses (Stumpo and Reinhold 2010; Gebrehiwot et al. 2018; Wang et al. 2019; Kawahara et al. 2021). Humans do not endogenously synthesise Neu5Gc due to an inactive cytidine monophosphate *N*-acetylneuraminic acid (Neu5Ac) hydroxylase (CMAH) enzyme, which converts CMP-Neu5Ac to CMP-Neu5Gc, and is inactive (Suzuki 2006). Despite this, exogenous Neu5Gc can be taken up and metabolically activated to CMP-Neu5Gc for incorporation into human glycoconjugates (Huang et al. 2026).

In human cancer tissue and secretion material, Neu5Gc has been reported at very low levels, particularly when compared to the more common sialic acid, Neu5Ac (Shewell et al. 2018; Shewell et al. 2022; Shewell et al. 2023; Wang et al. 2023; De Bisscop et al. 2026). Despite the clear association of Neu5Gc with cancer tissue, and the serum of people with cancer, the very low abundance of Neu5Gc has hampered progress towards developing a specific and accurate biomarker, with a specific Neu5Gc lectin only having been developed recently (Wang et al. 2018) and a polyclonal chicken IgYanti-Neu5Gc reagent (Diaz et al. 2009). While anti-glycan reagent specificity can at times be unparalleled, more direct evidence such as that provided by mass spectrometry (MS) is often required for further confirmation.

In part, the lack of development of a Neu5Gc related disease biomarker reflects the limited sensitivity in available instrumentation to detect the presence of low abundance Neu5Gc in human samples. Determination of relative limits of detection for lowly abundant molecules can have direct benefits for downstream biomarker detection and validation, potentially leading to more specific disease diagnosis and treatment (Zhang et al. 2019). Mass spectrometry-based analytical methods for glycomics can be performed in a multitude of ways, including as glycopeptides or glycolipids, and as released glycans, both labelled and unlabelled (Fan et al. 2025).

Usually, quantitative sialic acid analysis is performed via 1,2-diamino-4,5-methylenedioxybenzene (DMB)-liquid chromatography (LC)-FLR (Sato et al. 1999), which involves acid hydrolysis of the glycan to release the sialic acids and subsequent labelling with the fluorescent molecule DMB. DMB-labelled sialic acids are typically analysed using a liquid chromatograph coupled to a fluorescence detector, however this method is also compatible with mass spectrometry detection (Omoto et al. 2025). Hydrolysis of glycans to their monosaccharide constituents is destructive and results in a loss of overall structural information and thus, biological context, but with the benefit of access to comprehensive standards. In contrast, released glycan analysis by MS has the potential to provide both structural and compositional information on glycans.

MALDI- and LC–MS can measure released, intact glycans, providing insight into how monosaccharides are linked together and reflecting overall biosynthetic pathways. Analysing intact structures can enhance specificity, for example by distinguishing Sialyl Lewis epitopes that contain both fucose and sialic acid linkages (Palmisano et al. 2013). While these methods generally provide relatively unbiased profiling of glycans, with some targeted assays reported (Ruhaak et al. 2018; Ashwood and Cummings 2025), untargeted analyses that quantify tens to hundreds of structures in a sample seldom evaluate the quantitative accuracy of these measurements.

To assess whether changes in glycan abundance measured by MS truly reflect changes in concentration, calibration curves are essential. Quantitative accuracy depends on whether an increase in analyte concentration produces a proportional increase in measured signal, with the lower limit of quantitation (LLOQ) defining the point below which this relationship no longer holds (Currie 1995). In glycomics, the relevant matrix depends on the sample and the glycan release method, and comprises both the glycans of interest and any co-eluting contaminants present during LC–MS injection. Matrix-matched calibration curves (MMCCs) have been widely adopted in other -omics fields to enable accurate, multiplexed quantitation. They are routinely applied in proteomics for thousands of peptides (Pino et al. 2020) and in metabolomics or environmental analyses for tens to hundreds of analytes (Kang et al. 2007). This approach has yet to be systematically applied to glycomics, despite its potential to improve quantitative reliability across multiple glycan compositions and structures.

By maintaining the total sample matrix while varying the target analyte systematically, the analyte is exposed to the same matrix effects at every concentration. This allows the relationship between analyte abundance and measured signal, under conditions representative of actual samples. As a result of these MMCCs, linearity, LLOQ, ULOQ, precision and bias can then be assessed. Distributing the same defined benchmark series across participating sites enabled direct comparison of platform- and workflow-specific analytical performance while controlling for variation in sample composition. Traditional calibration methods remain limited by the availability and cost of isotopically labelled internal standards (e.g. ^13^C-labelled Neu5Gc glycans), so matrix-matched dilution strategies offer an attractive route to assess quantitative accuracy in complex glycan mixtures across LC–MS platforms.

We hypothesise that there has been an under-reporting of Neu5Gc related glycan structures in MS data from human glycomic samples, due to a combination of restrictive search spaces in data analysis, variation of analytical sensitivity across mass spectrometers, and low levels of abundance of Neu5Gc containing glycans. To assess the detection and quantitation limits of released glycans, with the goal of determining sensitivity towards Neu5Gc in human samples, we provided a set of samples to multiple independent MS labs with well-established routine glycomics workflows. These samples contained initially evaluated matrix matched standards of reduced *N*-glycan alditols carrying Neu5Gc containing glycans in a serial dilution. Appropriate analysis of these standards allows for a matrix matched calibration curve to be developed (Pino et al. 2020), determining limits of detection (LOD) and LLOQ for Neu5Gc containing *N*-glycans against a complex background. To the best of our knowledge, this is the first time this has been applied to analyses of released *N*-glycans. Conclusions drawn from this study provide insights into instrumentation-specific sensitivity limits, contextualizing platform-specific findings. A comparative study was also performed against the traditional labelled DMB-LC-FLR analysis, providing information on differing levels of sensitivity with a matched sample set.

## Results

### Experimental design

*N*-glycans enriched in Neu5Gc were released and profiled from a proprietary source. These *N*-glycans were predominantly Neu5Gc-modified, and were primarily biantennary, complex class (Fig. 1A). Due to these characteristics, this sample was suitable as the Neu5Gc target glycome for the MMCC (Fig. 1B). As MMCCs require a closely matched matrix containing minimal endogenous target relative to the calibration additions, human serum *N*-glycans were chosen as a matrix because they are structurally similar (complex, biantennary) and contain only trace amounts of Neu5Gc (Fig. 1C).

**Figure 1:**
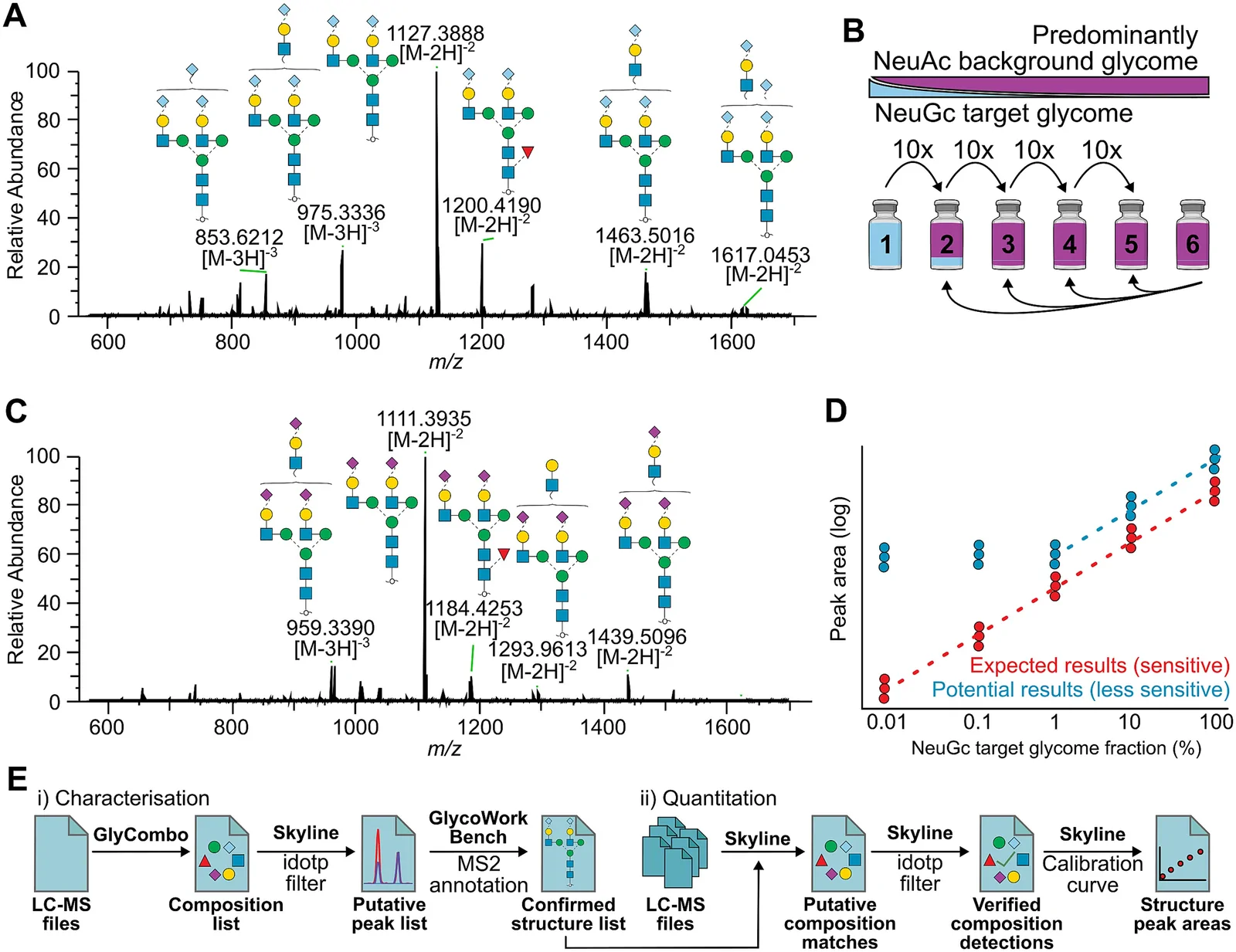
Development and application of a matrix-matched calibration curve to quantify Neu5Gc-bearing *N*-glycans. A) Neu5Gc enriched sample *N*-glycan profile (tube 1). B) Design of the Neu5Gc benchmark set. C) Human serum *N*-glycan profile to be used as the background matrix. D) Expected results when analysing the Neu5Gc benchmark set. E) Data analysis workflow for the multi-site study.

To develop a set of samples for benchmarking Neu5Gc detection across multiple sites, a total of six samples were generated by serially diluting the Neu5Gc target *N*-glycome with the Neu5Ac background *N*-glycome, covering four orders of magnitude dynamic range (Fig. 1D). A multi-site laboratory study was initiated in which each set of six tubes (the benchmark set), prepared at Site 1, was analysed. Each participating group was sent at least one benchmark set for oligosaccharide analysis by LC–MS and/or monosaccharide analysis by LC-fluorescence (FLR).

Data were collected from each site (for a total of 4 LC–MS instruments) and subjected to a data analysis workflow (Fig. 1E) to obtain qualitative and quantitative values for each glycan observed. To build the glycan list for assessing identification and quantitation quality at each Neu5Gc concentration, GlyCombo (Maia Kelly 2024) was used to identify glycans subjected to MS2, with Skyline (MacLean et al. 2010; Adams et al. 2020) subsequently used to filter for high quality glycan identifications with isotopic distributions matching expected. Structure was then verified by MS2 annotation in GlycoWorkbench (Ceroni et al. 2008). This list of glycans was then used as the target list within Skyline, with isotopic distribution filtering only performed to maintain quality before extracting glycan peak areas.

### Qualitative comparisons for Neu5Gc detection

Neu5Gc glycans can be distinguished from their Neu5Ac counterparts using several independent measurements across the PGC-LC–MS workflow. At the MS1 level, in addition to the difference in mass (291.10 vs 307.09 Da), both the monoisotopic precursor mass and the approximate ratio of the naturally occurring isotopes (^15^N, ^13^C, ^2^H, ^18^O) can be predicted, enabling comparison between the observed isotopic distribution and the theoretical distribution. The idotp metric (with 1 being a perfect match) can thereby confirm that an observed peak corresponds to the expected glycan chemical formula, and can distinguish true glycan signal from chemical and/or detector noise.

Since MS2 is triggered on a single precursor *m/z*, complementing it with the predicted isotopic distribution allows the full MS1 information in each raw file to be assessed, limiting the impact of noise on detection. As expected, idotp values were highest for the Neu5Gc-enriched sample analysed without background matrix and decreased with decreasing concentration, with values across almost all LC–MS platforms inversely correlated with dilution factor (Fig. 2A).

**Figure 2:**
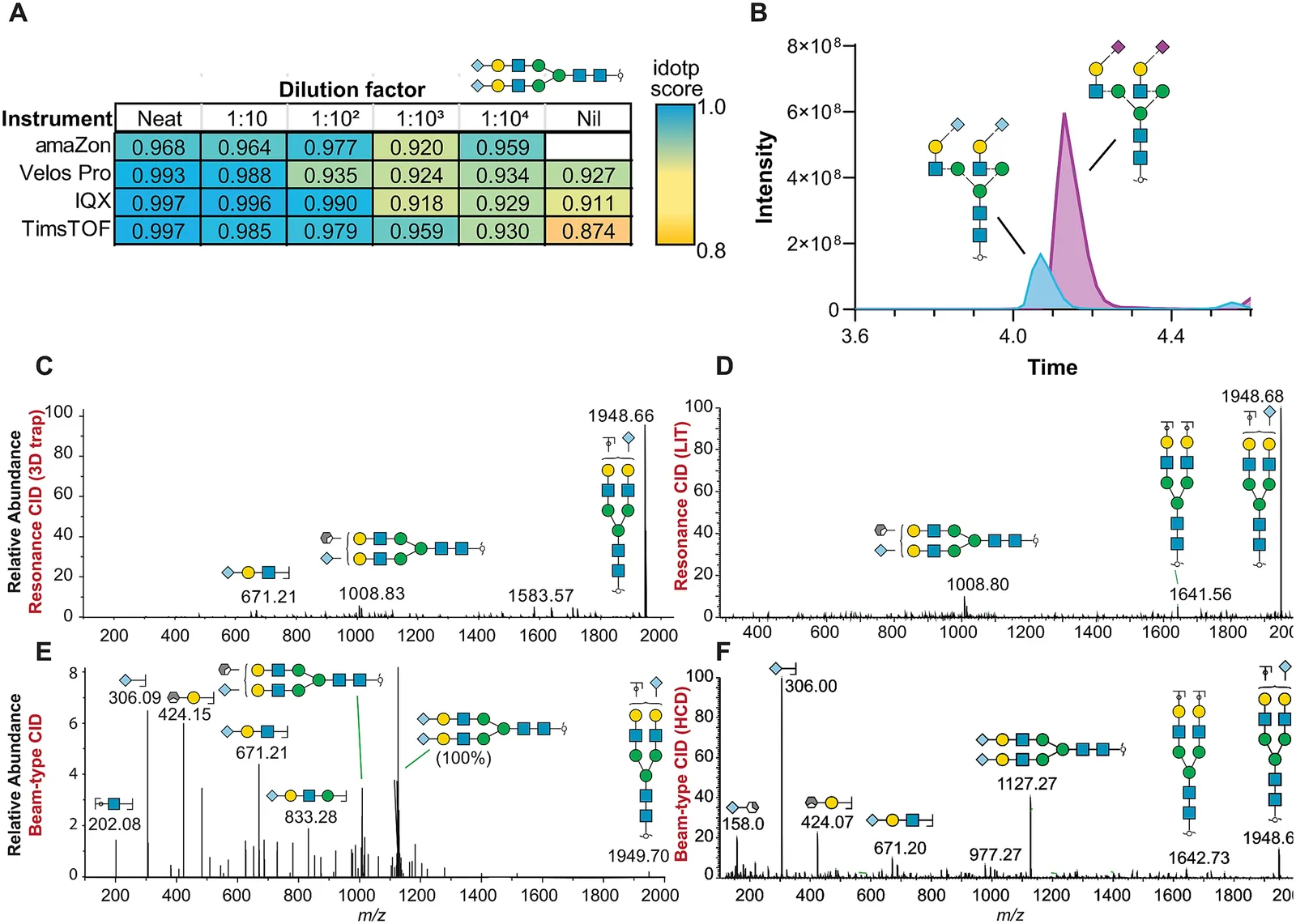
Detection and fragmentation of Neu5Gc biantennary glycan. A) Average idotp of biantennary Neu5Gc glycan from all participating instruments according to dilution factor. B) Partial co-elution of Neu5Gc and Neu5Ac variants of the same glycan. Chromatographic trace generated with data from site 1. Different fragmentation methods from different instruments result in varying fragmentation spectra, as exemplified by resonance 3D trap CID (C), linear ion trap resonance CID (D), beam-type CID (E), and HCD (F) data shown.

Chromatographically, Neu5Gc glycans consistently elute ahead of their Neu5Ac counterparts on the PGC, a separation best resolved at a 1:10 dilution factor. This separation matters for targeted analysis, as the two species partially overlap and are therefore prone to mutual interference and ion suppression (Fig. 2B). Neu5Gc glycans also display a defined ion mobility profile that can be exploited to further separate them from Neu5Ac (Supplementary Fig. 2).

At the MS2 level, fragmentation behaviour is technique-dependent. Resonance collision induced dissociation (CID), as used on the amaZon (Bruker) and Velos Pro (ThermoFisher) instruments, produces no detectable Neu5Gc monosaccharide signals due to the 1/3 rule that applies to ion trap instruments (March 1997; Ashwood et al. 2017); one of the smallest product ions observed is 671.22 *m/z* ([M-H]^−^) while charged losses of Neu5Gc from the precursor can also be observed (Fig. 2C–D). In contrast, beam-type CID generates the Neu5Gc monosaccharide at 306.08 *m/z* as the most abundant product ion, except in the timsTOF Pro data where the unfragmented precursor dominated (Fig. 2E–F); additional fragments such as Hex+Neu5Gc are informative and detectable across all platforms.

Because the fragmentation pattern depends on the dissociation method, search strategies must be tailored accordingly. Diagnostic-fragment approaches that rely on the Neu5Gc monosaccharide, such as that of Helm et al. (Helm et al. 2021) are unsuitable for resonant CID, where the low-mass cut-off (approximately one-third of the precursor *m/z*) prevents detection of the monosaccharide, and spectral library-based searching is likewise fragmentation method-specific (Helm et al. 2021).

### Quantitative comparisons for Neu5Gc detection

A calibration curve, as illustrated in Fig. 3A, serves to define the quantifiable range, bounded by the lower (LLOQ) and upper (ULOQ) limits of quantitation, corresponding to noise and saturation respectively. Variation, represented by the difference in intensity between replicates at the same concentration, ideally shows less than 20% coefficient of variation.

**Figure 3:**
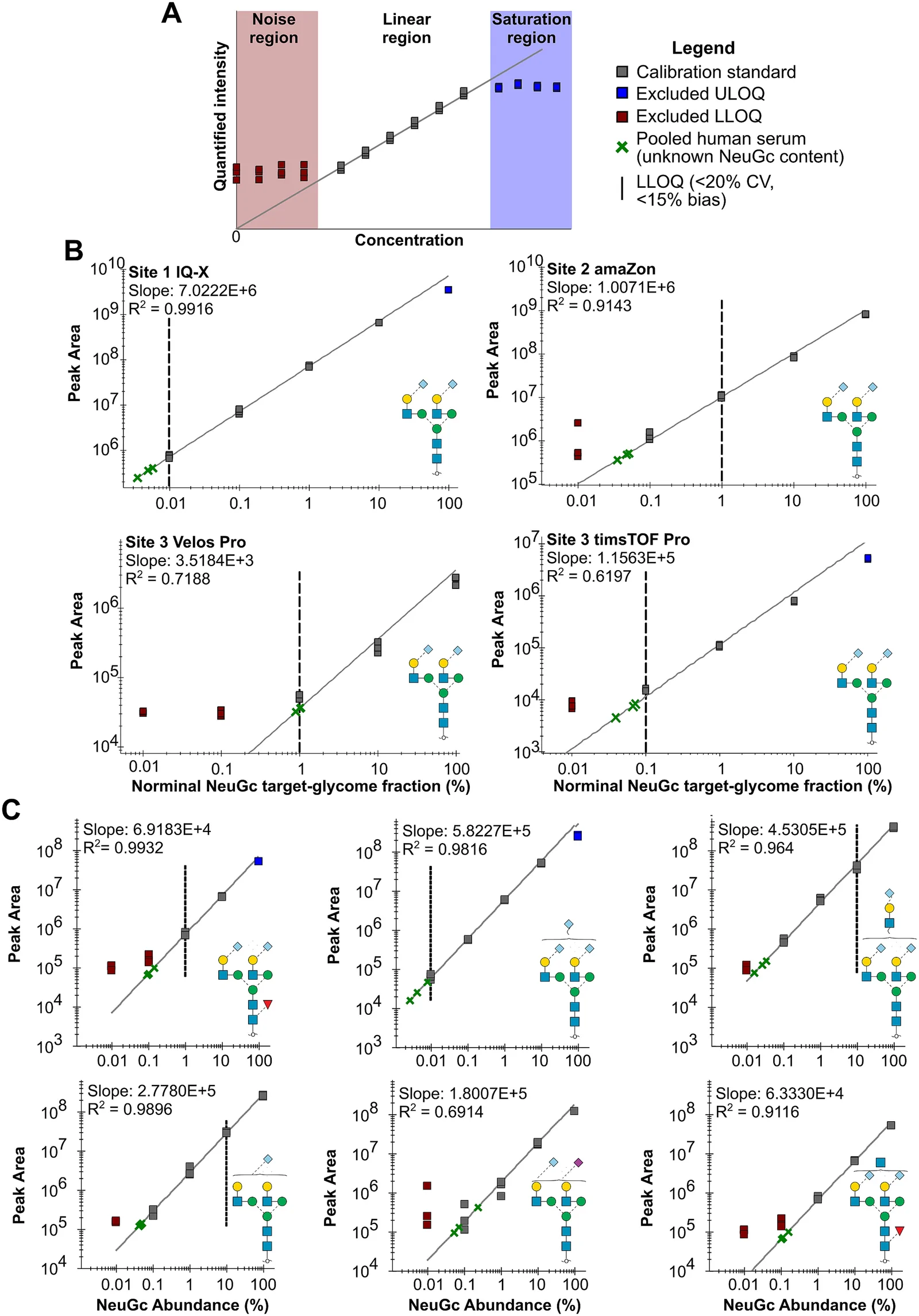
Quantitative assessment of Neu5Gc benchmark standards reveal instrument and structure-specific variation and accuracy. A) Terminology guidance for a calibration curve. B) Site comparisons for calibration curves of the most abundant Neu5Gc-bearing *N*-glycan. C) Calibration curve comparisons for structures acquired at site 1.

In the case of Site 1 (IQ-X, Fig. 3B), the most concentrated sample saturated the MS, and was therefore outside the linear range, above the ULOQ, while the remaining concentrations were above the LLOQ. Comparing the most abundant *N*-glycan across the remaining sites, various reasons were responsible for the LLOQ being higher, such as variation at low concentrations (Site 2 amaZon), or loss of linearity (<15% bias, Site 3 Velos Pro). As expected, newer instruments leveraging more recent advancements (IQ-X and timsTOF Pro) showed lower LLOQs in this comparison. Site 1 served as the benchmark for assessing dynamic range. By injecting the largest amount of material, it was the only site to detect Neu5Gc-bearing *N*-glycans in neat human serum, below the intensity of the lowest-concentration mixture, at up to 0.010% of total sialylation. Extrapolation of the linear regression constrained through zero yielded an estimated target-glycome equivalent of 0.0047 ± 0.0012% (mean ± SD), although this value lies below the validated quantitative range. At all other sites, the human sample was indistinguishable from noise.

Moving beyond the biantennary, disialylated *N*-glycan, other structures were quantified across the sites, each at a lower intensity and therefore higher LLOQ (Fig. 3C). The second most intense Neu5Gc glycan, a trisialylated biantennary *N*-glycan, had a similar calibration curve to the most abundant glycan. For less intense glycans, loss of linearity and greater variation caused the LLOQ to be higher. Thus, LLOQ values are analyte-specific, related to abundance, rather than based on a single property of the instrument.

For the past decade, the gold-standard method for analysing sialic acids has been LC-FLR of fluorescently labelled monosaccharides, an example workflow of which is shown in Fig. 4A. This approach has been widely adopted due to its selectivity for sialylated monosaccharides (Bardor et al. 2005, Martin et al. 2005, Samraj, et al. 2014, Samraj, et al. 2014, Ye et al. 2020). *O*-acetylated sialic acids, noted in non-human mammalian samples (Ashwood 2026), were not observed due to the prior alkaline reduction steps employed which degrade labile *O*-acetyl modifications (Jensen et al. 2012; Moh et al. 2024). By calibrating unknown sialic acid concentrations with a standard curve of known sialic acid concentrations, the absolute amount of an observed monosaccharide can be calculated based on the defined relationship between amount and fluorescence intensity, as demonstrated in Fig. 4B.

**Figure 4:**
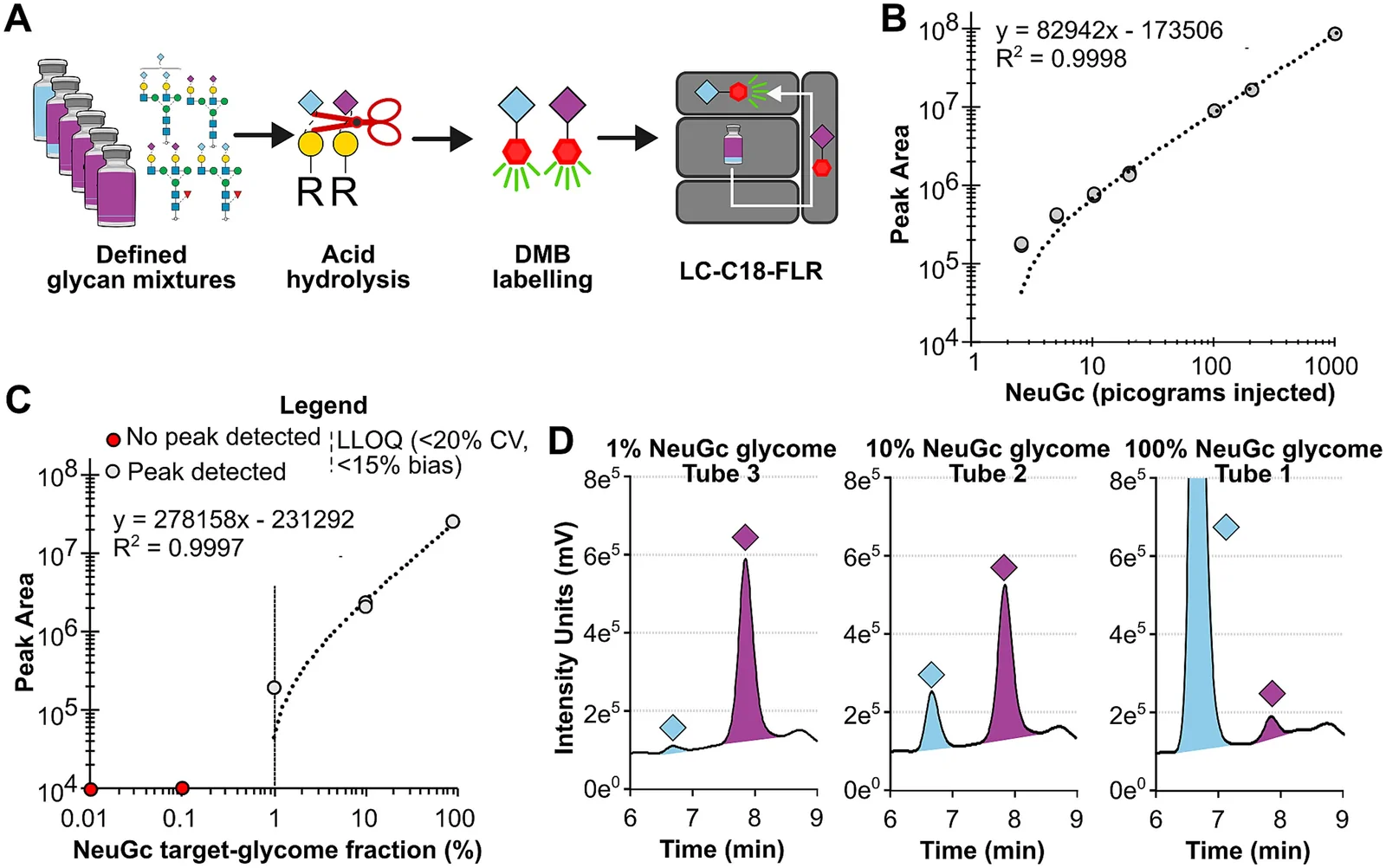
Dynamic-range limitations of DMB-LC-FLR in the defined Neu5Gc benchmark series. A) Monosaccharide analysis workflow was applied to Neu5Gc benchmark standards. B) Absolute monosaccharide standard calibration curve reveals linearity across two orders of magnitude. C) Two orders of magnitude dynamic range was insufficient to reliably detect Neu5Gc in pooled human serum. D) Example chromatogram of a Neu5Gc and Neu5Ac mixture at the limit of detection.

When this method was applied to the benchmark Neu5Gc sample set previously characterised by LC–MS, a peak corresponding to Neu5Gc was detected in the three most abundant samples (Fig. 4C). In contrast to LC–MS, where signal was detected but variable beyond 20% coefficient of variation at most sites, the Neu5Gc peak for the remaining three samples could not be confidently detected, owing to the consistent background noise inherent to the assay. The monosaccharide analysis results also reflect the limitations of the assay with the 1:100 dilution factor sample barely registering a Neu5Gc peak (Fig. 4D). Importantly, this approach also provided absolute concentrations of both Neu5Ac and Neu5Gc monosaccharides within the DMB quantitation range across the benchmark samples (Supplementary Table 1), contextualising the concentration ranges that defined the sensitivity and quantitative limits of both LC–MS and LC-FLR assays.

## Discussion

### Analytical platforms vary in their Neu5Gc detection capabilities

The multi-site benchmarking study revealed clear LC–MS-specific differences in the LLOQ for Neu5Gc containing *N*-glycans, emphasizing that the conclusions drawn regarding the presence of Neu5Gc in human samples are inherently platform-specific. Only Site 1 was able to detect Neu5Gc-bearing *N*-glycans in neat human serum, at ~0.0047% total sialylation by extrapolation below the validated LLOQ. On the other platforms employed in this work, this level was consistent with noise. Each site injected a different amount of *N*-glycans, however, with exception to the timsTOF, the differences in dynamic range exceeded what the 10–100× differences in injected material could account for.

Idotp values deteriorated with increasing dilution, confirming that MS1 isotopic distribution is a reliable proxy for signal confidence at low abundance and should be used as a routine quality metric in Neu5Gc detection workflows. This approach does have its limitations as at one site, significant carryover was observed, which emphasises the importance of block randomization and/or re-injection after blank acquisitions to limit the effect of carryover on false detections. Idotp measurement itself also has limitations, and cannot discriminate isomeric compositions, so orthogonal methods and metrics (e.g. MS2 and fragmentation coverage) are needed to resolve these interferences.

Fragmentation of *N*-glycans was, as expected, also platform-specific, with resonant CID instruments (amaZon, Velos Pro) failing to generate the diagnostic monosaccharide product ion for Neu5Gc, 306.08 *m/z*, which was observed on beam-type instruments (timsTOF Pro, IQ-X). Despite this, the neutral and charge losses of Neu5Gc were observed across all platforms, as well as larger product ions containing Neu5Gc (e.g. 671.22 *m/z [M-H]^−^*). In the untargeted methods used here, fragmentation of *N*-glycans of interest is not guaranteed, but the use of targeted methods (e.g. PRM/tMS2) may offer a solution for instruments where noise overwhelmed signal, given the specificity of precursor and product-ion targeting.

### The limitations of gold-standard LC assay for sialic acid content

Comparison between LC–MS and the current gold-standard DMB-LC-FLR approach (Manzi et al. 1990; Martín et al. 2007) demonstrated that released monosaccharide analysis could only detect Neu5Gc across two orders of magnitude, whereas LC–MS extended detection to three or more orders of magnitude despite measuring each Neu5Gc-containing structure individually. This suggests that LC–MS offers superior specificity and sensitivity for intact Neu5Gc-modified *N*-glycan detection at the cost of more expensive equipment and more complex data analysis. Additionally, the total Neu5Gc measurement after hydrolysis loses the monosaccharide origin context, preventing these absolute concentrations to be applied to intact Neu5Gc bearing glycans.

The two-order of magnitude dynamic range of the DMB-LC-FLR approach proved insufficient to detect Neu5Gc at concentrations representative of human serum, contextualising why these assays have largely been unable to detect Neu5Gc in clinical human samples. The absolute quantitation afforded by the DMB standard curve was valuable in this context as it provided the real concentration of the monosaccharides, unattained by the LC–MS approach used here. A hybrid strategy combining the two, such as LC–MS of released monosaccharides, may be a natural next step for subsequent analysis. In principle, this hybrid technique could yield absolute concentrations (using stable-isotope-labelled monosaccharide standards) while retaining the specificity provided by MS.

### Opportunities for Neu5Gc as a biomarker

Detection of Neu5Gc in human serum at only one site, at trace levels (~0.0047%) supports the hypothesis that Neu5Gc has been systematically under-detected in human glycomic datasets. This likely can be attributed to a combination of inadequate instrument sensitivity and overly restrictive search spaces, where Neu5Gc can be left out of data analysis workflows due to isomeric complexity and a subsequent increase in false positives (Klein et al. 2024; Bienes et al. 2025), and the absence of a rigorous LLOQ evaluation as performed here.

The analytical approach used here establishes the performance of LC–MS for measuring intact Neu5Gc-bearing glycans within defined mixtures, rather than assessing biological perturbations in Neu5Gc abundance arising from disease or inter-individual variability. Future validation should therefore examine biological variability across individual samples. Because the present study used pooled human serum, it provides only a single composite measurement, and the typical distribution and range of Neu5Gc abundance across the human population remain to be established. Because Neu5Gc abundance also varies across mammalian species in which it is more prevalent, including bovine, porcine, and canine systems, experimentally controlled animal-model studies could provide a useful means of evaluating assay responsiveness to biological variation and the generalisability of this analytical framework beyond the current benchmark.

Future studies evaluating Neu5Gc in human samples (whether across populations or in sample types beyond sera) should prioritise pairing this low abundance glycan epitope with high-sensitivity platforms (including antibody or mass spectrometry-based) rather than the DMB-LC-FLR approach, which is fundamentally unable to specifically detect or quantify Neu5Gc at concentrations found in neat pooled human sera with the method and sample loading evaluated here.

## Conclusion

This study applied a matrix-matched calibration curve to released *N*-glycans for the first time, providing a reusable framework for benchmarking Neu5Gc detection across LC–MS platforms and sites. Within this framework, LC–MS resolved Neu5Gc-bearing *N*-glycans over three or more orders of magnitude and, at the site with the largest linear range, detected Neu5Gc in neat human serum at ~0.01% of total sialylation, whereas the gold-standard DMB-LC-FLR assay was confined to two orders of magnitude and could not detect Neu5Gc at concentrations representative of human serum. These platform-specific limits strongly support that Neu5Gc has been under-detected in human samples due to its low abundance, and indicate that future studies should pair this low-abundance epitope with high-sensitivity LC–MS, ideally combining released-monosaccharide MS with isotopically labelled standards to recover absolute quantitation.

## Materials and methods

### Development of standard mixtures

All chemicals and reagents were purchased from Millipore Sigma (Castle Hill, Australia) unless specified otherwise. *N*-glycans were released from human serum (H4522-20ML, Sigma-Aldrich), and a proprietary Neu5Gc-rich glycoprotein mixture (Protea Glycosciences), as described in SUGA (Ashwood et al. 2025) with minor modifications. Briefly, approximately 1 mg of each protein mixture had cysteines reduced and alkylated, followed by protein precipitation by acidified methanol on DNA miniprep columns (Bioneer) (Mousseau et al. 2023). After precipitation, protein was resuspended in 240 μL of 100 mM triethylammonium bicarbonate with 200 units of PNGase F (Promega) and held at 37 °C for 18 h. This preparation was repeated in quintuplicate and in duplicate for the human serum and Neu5Gc rich material, respectively.

Following *N*-glycan release, *N*-glycans were eluted with 400 μL of 0.1% formic acid and then immediately dried by centrifugal evaporation. Once dry, *N*-glycans were reduced and desalted as described (Jensen et al. 2012; Moh et al. 2024), with 1 M NaBH_4_ in 50 mM KOH at 50 °C for 3 h. After reduction, *N*-glycans were desalted by Supelclean ENVI-Carb solid phase extraction (100 mg) with 400 μL acetonitrile/0.1% formic acid conditioning, 1.5 mL water/0.1% formic acid equilibration, sample loading, 1.5 mL water/0.1% formic acid desalting, and 400 μL 50:50 acetonitrile: water with 0.1% formic acid elution. Eluted, desalted glycans were then dried by centrifugal evaporation.

Each sample replicate was analysed by LC–MS, observed intensity of the most abundant glycan was recorded, pooled by sample identity, and diluted to be at the same approximately MS intensity across two final tube pools. *N*-glycan mixtures were made of the two components, with the Neu5Gc *N*-glycans serially diluted by Neu5Ac *N*-glycans 10-fold to form a total of 4 samples, for a total of 6 samples when including the Neu5Ac and Neu5Gc starting material as separate samples. Each tube contained an *N*-glycan equivalent of a release from 500 μg of protein. *N*-glycans were then dried and mailed at room temperature with receipt within 5 days. A total of 15 benchmark sample sets were generated within a single batch.

### LC–MS data acquisition methodologies

Initial characterisation by LC–MS was performed at Site 1. Glycans were separated with a Thermo Fisher Scientific Vanquish Horizon HPLC (San Jose, USA) and ionised into an Orbitrap IQ-X Tribrid mass spectrometer (San Jose, USA). A Thermo Fisher Hypercarb porous graphitised carbon (PGC) column (Lithuania, 100 mm length by 1 mm internal diameter, 3 μm particle size), held at 90 °C, was used for all separations. Mobile phase A composed of water and mobile phase B composed of acetone with 5 mM HFIP and 5 mM butylamine added.

Three sites (four different instruments) were sent sets of the benchmark standards with instructions (fully detailed in the Supplement). Briefly, glycans were supplied dry and the only variables between sites were the resuspension volume and buffer.

Each laboratory group’s specific approach is summarized in the Supplement, with chromatography and mass spectrometry equipment and parameters in Supplementary Table 2. Overall, the following methodology was consistent: Reduced *N*-glycans were separated on a porous graphitised carbon column over a binary gradient with the eluent subsequently analysed online by a mass spectrometer operated in negative mode, obtaining both MS1 (precursor) and MS2 (product) spectra.

### LC–MS data analysis

Upon acquisition, raw files were sent to the corresponding authors for subsequent analysis in Skyline-daily, using an assay template developed by Site 1. Spectrum visualization was performed with Freestyle 1.8 SP2 (Thermo Fisher Scientific), FlexAnalysis 3.4 (Bruker), and DataAnalysis 6.2 (Bruker). LC–MS raw files were analysed using GlyCombo (v1.2) to assign glycan compositions to precursor *m/z* values, identify the most intense MS2 scans for each structure for annotation in GlycoWorkbench, and construct the Skyline assay detailing *N-*glycan compositions. For Site 1, mzML input was used with an error tolerance of 25 ppm, reducing end specified as free, derivatisation as native and adducts set as M-H^−^. Monosaccharide search space was: Hex 4–12, HexNAc 2–8, dHex 0–1, Neu5Ac 0–3, Neu5Gc 0–3.

Skyline-daily (v25.1) was used to integrate the first three isotopic peaks with mass analyser set to centroid at 15 ppm mass accuracy. These isotopic integrations were used to quality filter identifications (> = 0.85 isotope dot product (idotp)) and quantify glycans. The idotp value of 0.85 was empirically selected to remove poor quality MS1-matches (caused by monoisotopic peak misassignment, incorrect charge assignment, and poor signal to noise ratios) while preserving high-quality matches. Quantitation was performed using a linear through zero regression fit, with no normalisation performed. Regression weighting was 1 / (x*x).

### Monosaccharide analysis sample preparation

An equal volume of 4 M acetic acid (Merck) was added to each sample. Samples were then incubated at 80 °C for 3 h to release sialic acids. Afterwards, 5 μL of each hydrolysed sample was added into 20 μL of 1,2-Diamino-4,5-methylenedioxybenzene dihydrochloride (DMB; Merck) labelling solution and then incubated at 50 °C for 2.5 h in the dark. DMB labelling solution contained Milli-Q water, 7.6% v/v glacial acetic acid, 0.74 M β-mercaptoethanol (Merck), 52 μM sodium hydrosulfite (Merck), and DMB at 1.6 mg/mL. Following this incubation, the DMB-labelled samples were diluted in 100 μL of ice-cold Milli-Q water. Samples were filtered using 0.22 μm syringe filters (Kinesis) and centrifuged at 900 × g for 5 min prior to analysis.

The DMB-labelled samples (Ex = 373 nm, Em = 448 nm) were then analysed on a Prominence-i HPLC (Shimadzu) using an XBridge Shield RP18 column (Waters) under isocratic elution in 86% Milli-Q water, 7% methanol, and 7% acetonitrile with chromatograms analysed with an intensity multiplier of 0.001. A Neu5Ac and Neu5Gc (Agilent) standard curve (Supplementary Fig. 1) and a DMB labelling solution only control were included in analysis.

## Supplementary Material

Neu5Gc_Manuscript_R2R1_Supplementary_cwag088

## Data Availability

Raw files are available on GlycoPOST (Watanabe et al. 2021) at https://glycopost.glycosmos.org/preview/15414602776a5429eb5a7fe with reviewer code 2328. Skyline assays and quantitation curves are available at Panorama (Sharma et al. 2014): https://panoramaweb.org/NeuGcBenchmark.url.
